# Look at Me: Early Gaze Engagement Enhances Corticospinal Excitability During Action Observation

**DOI:** 10.3389/fpsyg.2018.01408

**Published:** 2018-08-09

**Authors:** Sonia Betti, Giovanni Zani, Umberto Granziol, Silvia Guerra, Umberto Castiello, Luisa Sartori

**Affiliations:** ^1^Dipartimento di Psicologia Generale, Università di Padova, Padova, Italy; ^2^Centro Beniamino Segre, Accademia Nazionale dei Lincei, Rome, Italy; ^3^Center for Cognitive Neuroscience, Università di Padova, Padova, Italy

**Keywords:** action observation, gaze, attention, TMS, complementary actions

## Abstract

Direct gaze is a powerful social cue able to capture the onlooker’s attention. Beside gaze, head and limb movements as well can provide relevant sources of information for social interaction. This study investigated the joint role of direct gaze and hand gestures on onlookers corticospinal excitability (CE). In two experiments we manipulated the temporal and spatial aspects of observed gaze and hand behavior to assess their role in affecting motor preparation. To do this, transcranial magnetic stimulation (TMS) on the primary motor cortex (M1) coupled with electromyography (EMG) recording was used in two experiments. In the crucial manipulation, we showed to participants four video clips of an actor who initially displayed eye contact while starting a social request gesture, and then completed the action while directing his gaze toward a salient object for the interaction. This way, the observed gaze potentially expressed the intention to interact. Eye tracking data confirmed that gaze manipulation was effective in drawing observers’ attention to the actor’s hand gesture. In the attempt to reveal possible time-locked modulations, we tracked CE at the onset and offset of the request gesture. Neurophysiological results showed an early CE modulation when the actor was about to start the request gesture looking straight to the participants, compared to when his gaze was averted from the gesture. This effect was time-locked to the kinematics of the actor’s arm movement. Overall, data from the two experiments seem to indicate that the joint contribution of direct gaze and precocious kinematic information, gained while a request gesture is on the verge of beginning, increases the subjective experience of involvement and allows observers to prepare for an appropriate social interaction. On the contrary, the separation of gaze cues and body kinematics can have adverse effects on social motor preparation. CE is highly susceptible to biological cues, such as averted gaze, which is able to automatically capture and divert observer’s attention. This point to the existence of heuristics based on early action and gaze cues that would allow observers to interact appropriately.

## Introduction

In humans, eye contact may signal an approaching intention from the gazer toward the perceiver, and it is critical for communication and social interactions (Kleinke, 1986; Senju and Johnson, 2009; Schilbach et al., 2013; Hamilton, 2016). An early sensitivity to noticing people’s eye region and gaze direction is already detectable in newborns, which prefer faces showing a direct gaze compared to faces with averted or closed eyes (Batki et al., 2000; Farroni et al., 2002). Eye contact is so relevant in social development that a failure to develop typical gaze behavior is one of the earliest signals of social disorders, such as autism (Baron-Cohen, 1995a; Hamilton, 2016). Since the ability to rapidly detect other’s gaze represent an advantage for the human species, a peculiar eye configuration together with dedicated mechanisms for gaze processing have evolved to allow for an easy detection of gaze direction (Kobayashi and Kohshima, 2001). In line with this, Baron-Cohen (1995b) hypothesized the existence of an eye-direction detector (EDD) in humans, specialized in computing eye-gaze direction. In social contexts, an agent’s gaze can provide a cue possibly influencing the orientation of attention in given portions of space (for reviews, see Emery, 2000; Langton et al., 2000; Frischen et al., 2007). Observing another’s averted gaze can trigger in the onlooker a rapid and automatic shift of attention toward the gazed direction (e.g., Driver et al., 1999; Langton and Bruce, 1999; Friesen et al., 2005).

Together with eye-gaze, also head and limbs movements may determine a shift of attention toward specific aspects of the environment. Prinsen et al. (2017) showed their participants video clips of a hand opening and closing in front of a face gazing toward or away with respect to the observers. Direct eye contact, compared to averted gaze presentation, enhanced direct matching in the observers’ hand muscles (Prinsen et al., 2017). Other studies specifically explored the relationship between grasping actions, gaze and attention (see Atkinson et al., 2017 for an extensive review), addressing how observing another’s gaze and grasping behavior influences our own actions (e.g., Castiello, 2003; Pierno et al., 2006; Letesson et al., 2015). For example, Letesson et al. (2015) presented participants video clips in which an actor looked at the camera, then directed his gaze toward one of two objects in front of him and grasped it. Participants were then requested to perform a reach-to-grasp action toward target objects of the same or different size compared to the one grasped by the actor. Gaze and action cues differently modulated speed and accuracy toward the target object, but the combined availability of both cues led to a more accurate action execution. The relation between an agent’s hand posture and gaze in a reach to grasp task has also been considered in the context of complementary actions. Complementary actions (from Latin *complementum*; i.e., that fills up) are a specific class of movements which differ, while interacting, with observed ones (Sebanz et al., 2006; Sartori and Betti, 2015; Sartori, 2016). In a study by Innocenti et al. (2012), participants were requested to reach and lift a bottle in the presence of an empty glass, while their movement kinematics was recorded. When a conspecific produced a complementary request gesture (i.e., holding the empty glass while displaying a direct gaze to the participant), their lifting action was significantly interfered by the newly activated motor program of pouring, so that the grasping action on the bottle was slowed down (Innocenti et al., 2012).

Here we capitalize on previous research on complementary actions probing the motor system by means of transcranial magnetic stimulation (TMS) coupled with electromyography (EMG) (for reviews see Sartori and Betti, 2015; Sartori, 2016). Seeing an actor in a frontal position with an open hand signaling a request near a salient object, strategically placed close to the participant, induces a modulation in the observer’s muscular activity that is consistent with the intention to accept the request (i.e., grasping the object) rather than with the tendency to resonate with the observed action. Complementary response preparation seems to be very prompt and time-locked to the kinematics of other’s movement. The *functional shift*, in particular, indicates the ability to untie the automatic tendency to mirror another’s actions in order to prepare a complementary response (Sartori et al., 2013). Notably, this effect is founded on the “readiness to interact” (i.e., the willingness to engage in socially meaningful situations; Di Paolo and De Jaegher, 2012), since an arrow cue pointing toward the object instead of the hand gesture does not produce the same motor activations (Sartori et al., 2011b). Along these lines, Betti et al. (2017) recently manipulated participant’s attention while they were presented with action sequences requiring (or not) an interactive response. In particular, videos of an actress grasping a spoon full of sugar, pouring some sugar into a mug next to her on a table and then stretching toward a mug out of her reach – but close to the participant – as to pour some left sugar in it, were presented. Diverting attention by means of an exogenous cue (i.e., a red dot) interfered with the mirroring of non-interactive actions, but did not affect complementary response preparation. Here we extended previous evidence by adopting a more ecological setting: we manipulated the shift of *other’s gaze* in a social context eliciting complementary responses. In a preliminary study (Experiment 1) we investigated whether observing a request gesture while the actor directs his gaze toward a salient target object or toward the observer (i.e., direct gaze) differently affects his/her corticospinal excitability (CE). We reasoned that if direct gaze increases the observer’s social engagement, then CE in the corresponding muscle should grow. On the contrary, if motor preparation increases when the observed gaze points to the object, this might suggest that object salience and action predictability are the crucial nodes of this effect. The correlation of this neurophysiological index (CE) with the subjective experience of involvement was also investigated. In Experiment 2 we slightly modified the paradigm. Participants observed the actor looking at them (direct gaze) and then directing his gaze to the salient object or away from it. In real life situations, gaze cues and body kinematics are critical in guiding an observer’s behavior in a context-dependent manner and their separation, as stressed by Reader and Holmes (2016), may have adverse effect on the validity of social interaction experiments. In Experiment 2, the combined manipulation of gaze and body cues, and the adoption of different TMS timings were intended to better unveil the mechanisms underlying social motor preparation.

## Experiment 1

In Experiment 1 we assessed CE modulations while participants observed an actor performing a request gesture while directing his gaze toward a salient target object or toward the observer (direct gaze). In order to correlate this neurophysiological index of motor preparation with the subjective experience of involvement, at the end of the session participants fulfilled a questionnaire to quantify their willingness to interact.

### Materials and Methods

#### Ethics Statement

The experiments were approved by the ethics committee of the University of Padua (N°1817), in accordance with the Declaration of Helsinki (Sixth revision, 2008). All participants gave their written informed consent and were financially compensated for their participation.

#### Participants

Thirty-three naïve volunteers (18 female and 15 male, aged between 21 and 30 years, mean age 23.6 years) took part in the experiment. All participants were right-handed, as assessed with the Edinburgh Handedness Inventory (Oldfield, 1971), with normal or corrected-to-normal visual acuity. They were all screened for TMS exclusion criteria and for neurological, psychiatric and medical problems (Wassermann, 1998; Rossi et al., 2009). A right-handed non-professional actor (male, 29 years old) was recruited for video-clips recording.

#### Experimental Stimuli

**FIGURE 1:**
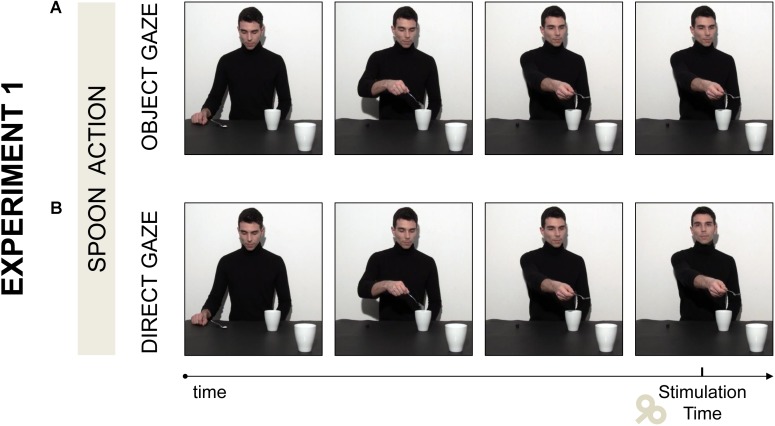
Two video clips were adopted in Experiment 1. **(A)** In the *Object gaze* video the actor stretched out his arm toward a mug out of his reach and looked at it. **(B)** In the *Direct Gaze* video the actor performed the same action, but at the end of the social request gesture he lifted his head and gazed toward the observer while his arm was stretched out. Single pulse TMS was delivered at the offset of the social request gesture.

Two video clips were adopted as experimental stimuli:

(a) *Object gaze*: the actor grasped a spoon full of sugar with a precision grip (PG; i.e., the opposition of the thumb to the index finger). Then he poured half sugar into a mug placed next to him and he finally stretched out his arm toward a mug out of his reach, with some sugar left in the spoon. The mug was strategically placed near the observer, in the right corner of the screen, thus affording a whole hand grasp (WHG; i.e., the opposition of the fingers to the palm) to be handled. The actor was instructed to follow his hand movements in a natural way with his gaze and *to look at the mug* while his arm was stretching toward it (**Figure 1A**).
(b) *Direct gaze*: the actor performed the same action as in the “Object gaze” condition, but at the end of the social request gesture he lifted his head and *gazed toward the observer* with his arm stretched out (**Figure 1B**).

Note that this social request would require the observer’s intervention to lift the mug and to complete the complementary action. This way, the observed movement (i.e., PG) was specifically mismatched with the one required to interact in a complementary fashion (i.e., WHG). Each video lasted 6210 ms and the animation effect was obtained by presenting a series of single frames each lasting 30 ms (resolution 1920 × 1080 pixels, color depth 32 bits) following the first frame lasting 500 ms.

#### Procedure

Participants were tested individually in a single experimental session lasting approximately 1 h and a half. They were seated in a comfortable armchair with the right arm positioned on a pillow and the head on a fixed head rest. They were instructed to remain as still and relaxed as possible while watching the video clips that were presented on a 24” monitor (resolution 1920 × 1080 pixels, refresh frequency 120 Hz) set at eye level (the eye-screen distance was 80 cm). No specific task was given to participants; however, they were told that at the end of the experiment they would be questioned about the stimuli presented (i.e., post-experiment questionnaire) to ensure attention to the video clips. TMS-induced motor-evoked potentials (MEPs) were acquired from the participants’ right first dorsal interosseous (FDI) and abductor digiti minimi (ADM) muscles of the right hand. A single TMS pulse was released during each video presentation at the end of the action sequence (5090 ms), namely when the actor’s arm was already stretched out toward the out-of-reach mug and his gaze was directed to the mug (Object gaze condition; **Figure 1A**) or to the observer (Direct Gaze condition; **Figure 1B**). The order of the video clips was randomized across participants. A total of 60 MEPs (2 muscles × 2 conditions × 15 repetitions) were recorded for each participant. Prior and after the experimental block, each participant’s baseline CE was assessed by acquiring 15 MEPs while they passively watched a white fixation cross on a black background presented on the computer screen. The average MEP amplitudes recorded during the two baseline periods (30 MEPs in total) was used to set each participant’s individual baseline for data normalization procedures. An inter-pulse interval lasting 10 s was adopted between trials in order to avoid any short-term conditioning effect (Classen et al., 2000). During this interval participants were reminded to remain fully relaxed for 5 s and a fixation cross was presented for the remaining 5 s. The presentation of a fixation cross before each trial ensured that participants always started the trial by observing the videos from a neutral gaze position. Stimuli presentation, timing of TMS stimulation and EMG recordings were managed by E-Prime V2.0 software (Psychology Software Tools, Inc., Pittsburgh, PA, United States) running on a computer.

##### TMS and EMG recording

Single-pulse TMS was administered using a 70 mm figure-of-eight coil connected to a Magstim BiStim2 stimulator (Magstim Co., Whitland, United Kingdom). Pulses were delivered to the hand region of the left primary motor cortex (M1). The coil was placed tangentially to the scalp, with the handle pointing laterally and caudally (Basil-Neto et al., 1992; Mills et al., 1992), in correspondence with the optimal scalp position (OSP) where MEPs with maximal amplitude were recorded simultaneously from the FDI and ADM muscles with the minimum stimulation intensity. To find the individual OSP, the coil was moved in steps of 0.5 cm until the position was reached. Once the OSP was found, it was marked on a tight-fitting cap worn by the participant. Then, the individual resting motor threshold (rMT), that is the lowest stimulus intensity at which TMS is able to generate MEPs of at least 50 μV in relaxed muscles in 5 out of 10 consecutive pulses (Rossini et al., 1994), was determined for the less excitable muscle (ADM). The stimulation intensity was then set at 120% of the rMT to record a clear and stable MEP signal throughout the experiment. rMT ranged from 32 to 56% (mean = 40% and SD = 5.1) of the maximum stimulator output. During the experimental sessions the coil was held by a tripod and continuously checked by the experimenters to maintain a constant positioning with respect to the marked OSP. MEPs were recorded simultaneously from the FDI and ADM muscles of the participant’s right hand. These muscles were chosen because of their differential activation during the observation of PG and WHG (e.g., Betti et al., 2018). In particular, ADM is only activated for WHG, whereas FDI is modulated during observation of both types of grasp (e.g., Gangitano et al., 2001). This aspect is crucial for the present manipulation, since we expect a muscular-specific activation for the ADM muscle when a request is made toward the large mug (i.e., a WHG) but not toward the small coffee cup, whereas the control muscle (FDI) should be activated in both cases. The EMG signal was recorded by means of two pairs of surface Ag/AgCl electrodes (1 cm diameter) placed in a belly-tendon montage, with the active electrode placed over the muscle belly and the reference over the interphalangeal joint. The ground electrode was positioned over the participant’s left wrist. Skin impedance, evaluated at rest prior to beginning the experimental session, was considered of good quality when below the threshold level (5 Ohm). Electrodes were connected to an isolable portable ExG input box linked to the main EMG amplifier for signal transmission via a twin fiber optic cable (Professional BrainAmp ExG MR, Munich, Germany). The raw myographic signals were band-pass filtered (10 Hz – 1 kHz), amplified prior to being digitalized (5 kHz sampling rate), and stored on a computer for off-line analysis. EMG data were collected for 300 ms after the TMS pulse by using Brain Vision Recorder software (Brain Products GmbH, Munich, Germany).

##### Post-experimental questionnaire

At the end of the experimental session participants were instructed to express on a five-point Likert scale (ranging from “Not at all” to “Very much”) their agreement or disagreement with four statements for each condition. The order of the conditions which the sentences referred to was counterbalanced between participants. Hereafter the adopted items (translated from Italian): (Q1) “I felt involved in the action”; (Q2) “At the end of the video I would have grabbed the nearest mug”; (Q3) “At the end of the video, I had the impression that the boy wanted to interact with me”; and (Q4) “At the end of the video I wanted to join the action”. These sentences were adopted to assess the experimental stimuli were effective in modulating perceived engagement and to quantify the subjective experience of involvement experienced by each participant during the experiment. One of the hallmark of our study was to explore the relationship between the participants’ perceived level of engagement and the corresponding CE modulations during video observation. To do so, we decided to cluster our sample in two groups of responders, in order to examine whether the different attitude toward the observed scenes (i.e., low and high engagement) was associated with different patterns of motor activations.

#### Data Analysis

Data were analyzed offline using Brain Vision Analyzer software (Brain Products GmbH, Munich, Germany) for EMG data and the softwares R (version 3.3.2; R Core Team, 2016) for statistical analysis. The MEP peak-to-peak amplitude (mV) for FDI and ADM muscles was determined as a measure of participants’ CE. Trials in which any EMG activity greater than 100 μV was present in the 100 ms window preceding the TMS pulse were discarded to prevent contamination of MEP measurements by background EMG activity. Trials contaminated by muscular pre-activation and values exceeding the 3 SD from the mean were excluded as outliers (<5%). Ratios were computed using the participant’s individual mean MEP amplitude recorded during the two baseline periods (MEP ratio = MEPobtained/MEPbaseline). Statistical analyses of the data were performed using a linear mixed-effects model on MEPs. Two predictors were used as fixed effects of the model, namely the Muscle (ADM and FDI) and Gaze direction (Object gaze and Direct gaze) predictors. The interaction between predictors was inserted in the model. Participants were set as a random factor (random intercept model), in order to assess the individual variability. The mixed models were performed using the R packages lme4 (Bates et al., 2014). The significance of the fixed effects was tested by means of a Wald Chi-Square test, performed by using the R package Car (Fox and Weisberg, 2011). A significance threshold of *p* < 0.05 was set for all statistical analysis. Each time a statistically significant effect was found, multiple comparisons were performed using the lsmeans package (Lenth, 2016). The degrees of freedom of such comparisons were computed using the Satterthwaite method, while the *p*-values were adjusted by means of the Tukey method (Gaylor and Hopper, 1969; Lenth, 2016). A mixed effects model was tested on the questionnaire scorings, with Condition (Object Gaze and Direct Gaze) and Item (Q1, Q2, Q3, and Q4) set as fixed effects, and subjects as random effect. Finally, to explore a possible link between the perceived engagement with the observed action and the observer’s CE, Pearson’s correlations between questionnaire scorings and MEP values were computed for each experimental condition and muscle. Bonferroni’s correction for multiple comparisons was applied to prevent Type-1 errors. Moreover, a cluster analysis was performed on the post-experimental questions running a k-means analysis. Two centroids for the final cluster membership configuration were set. The choice of selecting two centroids (groups) was taken in order to create two theoretical groups of responders: High and Low responders. These two groups were then used as a between factor for simple t tests to compare MEP scores between groups.

### Results and Discussion

**FIGURE 2:**
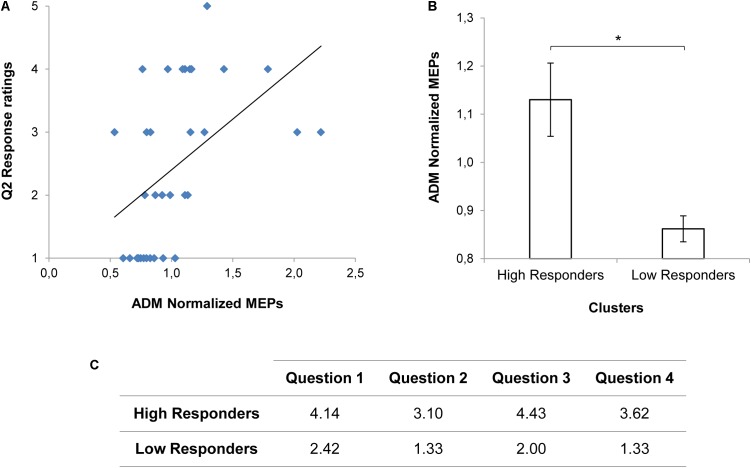
MEP values and Questionnaire scorings for Experiment 1. A positive correlation between the reported tendency to grab the nearest mug at the end of the observed action (Q2) and ADM MEP values for the Direct Gaze condition is shown in panel **(A)**. ADM MEP values for the High and the Low Responders in the Direct Gaze condition are graphically represented in panel **(B)**. Asterisk indicates a statistically significant difference (*p* < 0.05). Bars refer to the standard errors. Mean scorings for each item of the Questionnaire on the basis of the clusters (High and Low responders) are reported in panel **(C)**.

The results of the mixed model on the questionnaire responses confirmed that adopted stimuli were effective in modulating perceived engagement. A main effect of Condition (*χ*^2^_(1)_ = 45.81, *p* < 0.001), Item (*χ*^2^_(3)_ = 27.72, *p* < 0.001) and a significant interaction between Condition and Item (*χ*^2^_(3)_ = 13.48, *p* < 0.01) were found (see also **Supplementary Table 1**). In particular, Direct Gaze compared to Object Gaze conditions increased the scoring for Items Q1 (“I felt involved in the action”; *t*_(224)_ = -4.51, *p* < 0.001) and Q3 (“At the end of the video, I had the impression that the boy wanted to interact with me”; *t*_(224)_ = -5.78, *p* < 0.001). No statistically significant effects on MEP data were found (*p_s_* > 0.05; see also **Supplementary Table 2**). Nonetheless, a positive correlation emerged for the Direct Gaze condition between the reported tendency to grab the nearest mug at the end of the observed action (Q2) and MEPs in the corresponding ADM muscle (*r*_(31)_ = 0.479, *p* = 0.020; **Figure 2A**). Indeed, when considering the two clusters of responders (see **Figure 2C**), we found higher ADM MEP normalized scores for the High Responders than for the Low responders in the Direct Gaze condition (*t*_(27.35)_ = 2.54, *p* = 0.017; **Figure 2B**). The more the participants felt involved in the action, the higher was the activation in the muscle required for the interaction. The presence of a consistent subgroup of Low Responders might have affected the neurophysiological effect on MEPs. Therefore we decided to deeply consider this issue in Experiment 2.

## Experiment 2

Experiment 1 was not effective in eliciting CE modulation. This was possibly due to some alternative explanations (or to their combination): (i) participants were not socially sensitive; (ii) gaze manipulation was ineffective; and (iii) TMS stimulation timing was inappropriate. In order to disentangle these variables, a new Experiment was conceived. First of all, the Reading the Mind in The Eyes Test (RMET), a well-recognized test of social sensitivity (Baron-Cohen et al., 2001) was administered to participants. Then, a new paradigm was adopted in order to better control the gaze manipulation: after pouring in the close mug/cup (Step A), the actor started his social request gesture (Step B) while displaying direct eye contact, and then he looked at the salient object. In two control conditions, the actor’s gaze was diverted from the action and directed toward a neutral part of the visual scene. This way, direct and averted gaze potentially expressed the request to interact or not, respectively (George and Conty, 2008; Senju and Johnson, 2009). Moreover, eye-tracking recordings were acquired during observation of all the action sequences to validate that our manipulation had an effect on observers’ gaze behavior. Lastly, in the attempt to reveal possible time-locked modulations, we thoroughly investigated Step B by tracking participants’ CE at both the onset (T_1_) and offset (T_2_) of the request gesture. As a further control, we introduced a double dissociation to test the muscle-specificity of the neurophysiological effect. Two different types of grasps were shown in the videos (i.e., a precision grip on a spoon or a power grip on a thermos) to elicit two different complementary motor responses in the observer (i.e., a power grip on a mug or a precision grip on a coffee cup). Since different types of grasp differently involve the examined muscles (i.e., FDI and ADM), we hypothesized that only the target object affording a WHG (i.e., the mug) would have specifically activated ADM muscle. Whereas the control muscle (FDI) should be activated in both types of grasp.

### Materials and Methods

#### Participants

Thirty-three individuals were recruited. The data from three participants could not be used in the analysis due to technical problems. Therefore, thirty participants (20 female and 10 male, aged between 19 and 29 years, mean 23 years) with the same characteristics of those who took part in Experiment 1 were included in the analysis.

#### Experimental Stimuli

**FIGURE 3:**
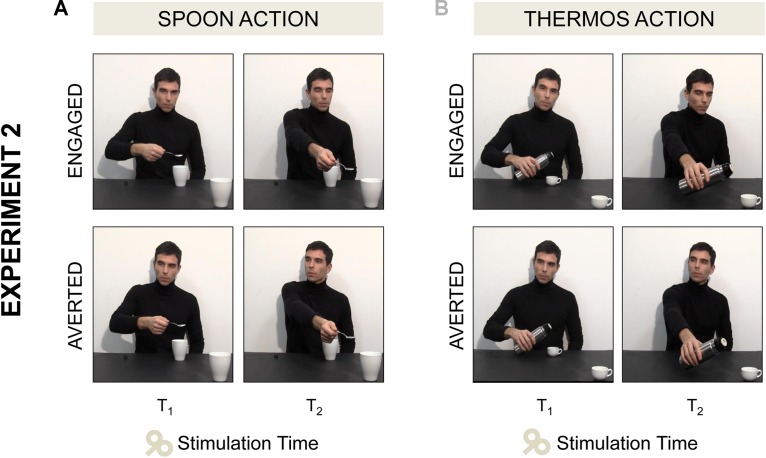
Four video clips were adopted in Experiment 2. **(A)** In the *Spoon-Engaged* video the actor started stretching out his arm toward a mug out of his reach while directing his gaze toward the observer (T_1_, see the first frame), then he completed the request gesture while gazing at the mug (T_2_, see the second frame). **(A)** In the *Spoon-Averted* video the actor performed the very same “Spoon” action, but when stretching out his arm toward the out-of-reach mug he turned away his gaze pointing to his right. **(B)** In the *Thermos-Engaged* and *Thermos-Averted* videos the same action sequences were shown, except for the type of grasp (WHG) and the target object (coffee cup).

Four video clips were adopted as experimental stimuli:

(a) *Spoon-Engaged*: an actor grasped a spoon full of sugar and poured some sugar into a mug placed next to him (Step A). Then he started stretching out his arm toward a mug out of his reach (Step B) while directing his gaze toward the observer (T_1_) and he completed the request gesture while gazing at the mug (T_2_; **Figure 3A**).
(b) *Spoon-Averted*: the actor performed the very same “Spoon” gesture throughout the video, but during Step B he started turning his gaze to his right (T_1_) and he completed the request gesture while gazing away (T_2_; **Figure 3A**)
(c) *Thermos-Engaged*: the actor grasped a thermos and poured some coffee into a coffee cup placed next to him on a table (Step A). Then he started stretching out his arm toward a cup out of his reach while directing his gaze toward the observer (T_1_) and he completed the request gesture while gazing at the cup (T_2_; **Figure 3B**).
(d) *Thermos-Averted*: the actor performed the very same “Thermos” gesture throughout the video, but during Step B he started turning his gaze to his right (T_1_) and he completed the request gesture while gazing away (T_2_; **Figure 3B**).

We specifically manipulated observers’ shifts of attention and social involvement by means of actor’s eye-gaze and head direction. The actor was instructed to keep looking at his hand until he had finished pouring (Step A) and then to unfold the social request action while performing either an interactive or an averted gaze (Step B). Notably, the type of grasp observed and the one required to respond to the social request were reciprocally mismatched in all the experimental conditions. All the videos underwent a *post-hoc* kinematic analysis using a digitalization technique (VideoTrack; Ab.Acus, Milano, Italy). Each movement was tracked by manually assigning a marker to the model’s wrist, nose and eyes. This analysis allowed excluding that any difference in terms of hand gesture was present across Engaged/Averted conditions, despite gaze and head movements differed. Notably, this procedure also allowed checking that gaze and head movements were always convergent. Each video lasted 9680 ms and the animation effect was obtained by presenting a series of single frames each lasting 40 ms (resolution 1920 × 1080 pixels, color depth 32 bits) following the first frame lasting 500 ms.

#### Procedure

Transcranial magnetic stimulation-induced MEP from the right ADM and FDI muscles were acquired during video observation. Eye-tracking measures were used to investigate overt attention allocation during the observation of the videos presented in the TMS session. Lastly, participants completed the Italian version of the RMET to evaluate their ability to attribute complex mental states from eyes’ observation.

##### Transcranial magnetic stimulation and electromyographic recording

The TMS-EMG procedure in Experiment 2 was identical to Experiment 1, with the following exceptions regarding the stimulation timing and the type of videos. TMS-induced MEP from the right ADM and the right FDI muscles were acquired once for each video presentation at one of two possible time points: (T_1_) when the actor’s wrist trajectory started to move toward the cup/mug next to the observer (onset of Step B; 5940 ms); (T_2_) when the actor’s arm was completely stretched toward the cup/mug near to the observer (offset of Step B; 7500 ms; see **Figure 3B**). Ten MEPs were acquired for each muscle (ADM and FDI), experimental condition (Spoon and Thermos), Gaze direction (Engaged and Averted) and Timing of stimulation (T_1_ and T_2_), resulting in a total of 160 MEP per participant (2 muscles × 10 repetitions × 4 video clips × 2 time points). rMT ranged from 29 to 51% (mean = 41%, SD = 6.1).

##### Eye tracking recordings

Eye movements were recorded by means of an infrared Tobii T120 Eye Tracker (Tobii Technology, Danderyd, Sweden), embedded in a 17” display. Eye position was sampled at 120 Hz with a spatial accuracy of 0.5 deg of visual angle. Prior to starting the experiment, the eye-tracker calibration was performed through a standard five-point grid, and repeated when necessary. Participants were seated at a distance of 65 cm from the monitor (1280 × 1024 pixels) and they were asked to observe the experimental stimuli (AVI format videos, 25 frames per second). Each trial started with the presentation of a fixation cross in the center of the screen and participants were instructed to look at the cross for 3 s. This ensured that all participants would start observing the video stimuli from the same origin point. Each video clip was presented to each participant three times in a randomized order, for a total of twelve presentations.

##### Reading the Mind in The Eyes Test (RMET)

The RMET (Baron-Cohen et al., 2001; Serafin and Surian, 2004) is a well-validated task used to investigate the ability to infer the mental states of others. It involves the presentation of 36 photographs of the eye region of male and female actors, flanked by one correct emotional term out of four. Participants were shown each photograph and asked to choose which word they thought best described what the person in the photograph was thinking or feeling. The total number of correct choices is indicative of the RMET performance. In addition, a gender control task, in which participants had to indicate the gender of the person depicted in the photograph, was administered to control for basic visual discrimination abilities.

#### Data Analysis

**FIGURE 4:**
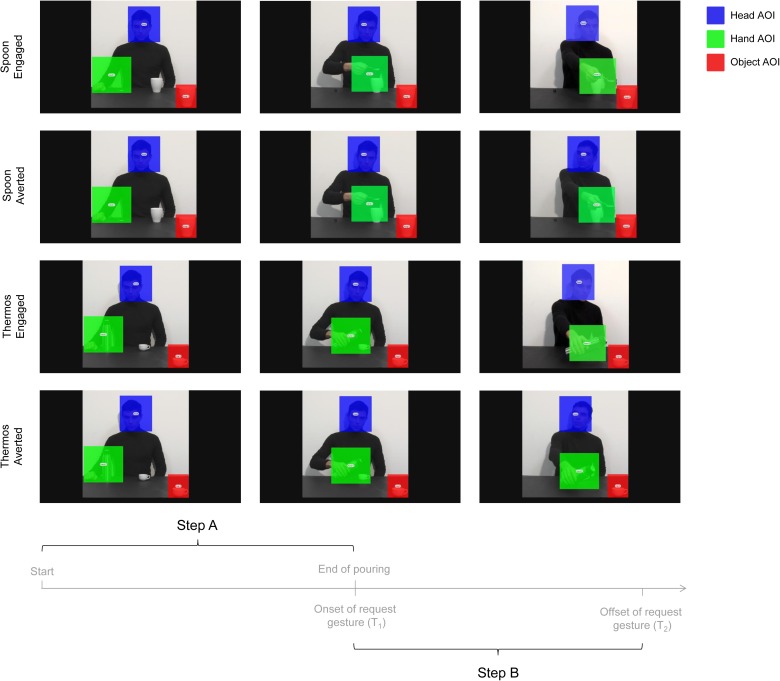
Sequence of events for the four experimental videos (i.e., Engaged and Averted conditions with Spoon and Thermos actions) and the time epochs considered for data analysis (i.e., Step A and Step B). The overlaid colored rectangular areas represent the adopted AOIs: Head AOI (blue); Hand AOI (green); and Object AOI (red).

Data for the RMET and the gender control test were analyzed to verify whether participants scored within the normal range. EMG and statistical analyses were performed as for Experiment 1, except that three predictors were used as fixed effects of the model – namely Muscle (ADM and FDI), Type of action (Thermos and Spoon) and Gaze direction (Engaged and Averted). Eye-tracking data were analyzed using Tobii studio 3.1 (Tobii Technology, Danderyd, Sweden) to investigate fixations targeted to specific regions of interest (i.e., AOIs; Areas of Interest). A fixation event was computed when gaze remained within 0.5 degrees of visual angle for at least 100 ms. For each video, three AOIs were adopted (see **Figure 4**): (i) Head AOI (203 × 225 pixels): a static area which included the actor’s head; (ii) Hand AOI (248 × 228 pixels): a dynamic area which included the actor’s hand while performing the action; and (iii) Object AOI (129 × 147 pixels): a static area covering the mug/cup placed near the observer, in the bottom right corner of the screen. Gaze behavior within the AOIs was measured for the entire duration of the video stimuli. Fixation Duration (i.e., the average duration in seconds for all fixations within the AOI) was specifically investigated during two temporal windows – namely Step A and Step B – preceding and following the start of the social request. The first temporal window included the entire actor’s action from movement onset to end of pouring in the close mug/cup (Step A; 6320 ms duration; **Figure 4**). The second temporal window, instead, comprised the start of the social request to the end of the video clip (Step B; 3360 ms duration; **Figure 4**). Statistical analyses of the data were performed using a linear mixed-effects model on eye-tracking measures.

### Results and Discussion

**FIGURE 5:**
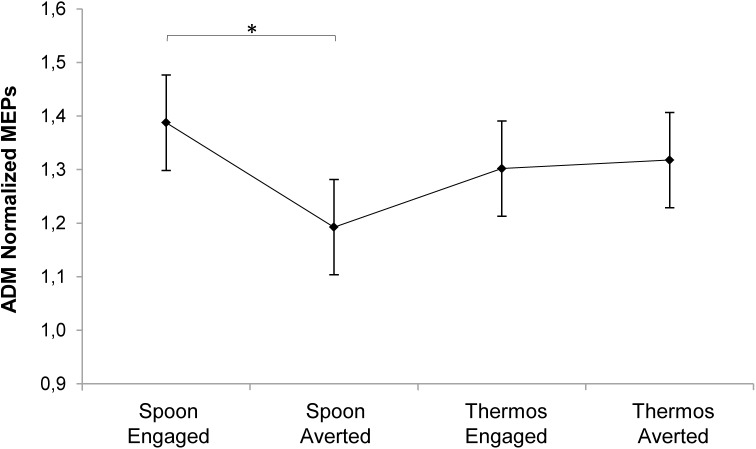
Plot of the interaction between action and condition predictors on MEP normalized amplitude for the ADM muscle at T_1_. Asterisk indicates a statistically significant difference (*p* < 0.05). Bars refer to standard error.

All participants scored within the normal range (cut-off = 13/36; see Baron-Cohen et al., 2001) both at the RMET test (26.67 ± 2.63) and at the RMET gender control test (35.17 ± 0.95), in line with normative data from an Italian sample of similar age with no previous history of mental disorders (*N* = 75, test score: 25 ± 3.9, gender control score: 35.4 ± 0.9; see Serafin and Surian, 2004). Please refer to **Supplementary Table 3** for descriptive statistics of normalized MEP. As concerns the mixed model performed on MEPs considering all the independent variables, the time predictor showed a statistically significant effect (*χ*^2^_(1)_ = 17.18, *p* < 0.001). MEPs at T_1_ (i.e., request gesture onset) were higher than those measured at T_2_ (i.e., request gesture offset; *t*_(435)_ = 4.15, *p* < 0.0001). This might explain the lack of results in Experiment 1, where the TMS pulse was delivered at the request gesture offset. The condition predictor also showed a significant effect (*χ*^2^_(1)_ = 8.75, *p* = 0.003). MEPs for the Engaged conditions were higher than for the Averted conditions (*t*_(435)_ = -2.96, *p* = 0.003). Splitting the overall model across the time predictor’s levels, at T_1_ both the condition (*χ*^2^_(1)_ = 8, *p* = 0.005) and the interaction between condition and action (*χ*^2^_(1)_ = 5.42, *p* = 0.02) were significant. MEPs at T_1_ for the Engaged conditions were higher than for the Averted conditions (*t*_(203)_ = -2.82, *p* = 0.005). Such a comparison was significant only for the Spoon action (*t*_(203)_ = -3.65, *p* = 0.002). Two separate mixed models were performed considering each muscle separately since the adopted intensity of stimulation based on the ADM threshold value may have over-stimulated the FDI muscle. In order to minimize this potential stimulation bias, we conducted the analysis separately for each muscle (see Cavallo et al., 2011 for a similar approach). For the ADM muscle, a statistically significant interaction emerged at T_1_ between action and condition predictors (*χ*^2^_(1)_ = 5.3, *p* = 0.02). MEPs for the Spoon-Engaged condition were higher compared to the Spoon-Averted condition (*t*_(87)_ = -3, *p* = 0.002; **Figure 5**). This indicates that ADM was specifically activated by a request upon the mug (i.e., affording a WHG) when the gaze was pointing to the observer rather than away. For the FDI muscle’s model, only a significant effect of the condition predictor emerged at T_1_ (*χ*^2^_(1)_ = 7.89, *p* = 0.005). MEPs for the Engaged conditions were greater than those for the Averted conditions (*t*_(87)_ = -2.8, *p* = 0.006). No significant effect resulted for FDI MEP amplitudes in terms of action predictor, since FDI was involved in the motor preparation of both complementary responses.

#### Eye-Tracking Results

Please refer to **Supplementary Table 4** for descriptive statistics of fixation duration. As the three AOIs differed in their dimensions, they were considered separately in the analysis for meaningful comparisons. The analysis on the first part of the action (Step A), when the action sequences were identical and no gaze manipulation was presented, led to no statistically significant effects. Conversely, significant effects emerged for all three AOIs (Hand, Head, and Object) during Step B, when the social request was expressed and gaze direction was manipulated (Engaged and Averted). In particular, the Hand AOI presented a significant effect of the condition predictor (*χ*^2^_(1)_ = 4.09, *p* = 0.042). The hand was observed for a longer time in the Engaged compared to the Averted gaze condition (*t*_(87)_ = -2.02, *p* = 0.046). After splitting such model across the action predictor’s levels, a significant effect was found for the Thermos action (*χ*^2^_(1)_ = 6.05, *p* = 0.014). The hand was observed for a longer time in the Thermos-Engaged compared to the Thermos-Averted condition (*t*_(29)_ = -2.46, *p* = 0.02). As concerns the Head AOI, in the overall model the action predictor presented a statistically significant effect (*χ*^2^_(1)_ = 4.02, *p* = 0.045). The Head was observed for a longer time during the Spoon rather than the Thermos action (*t*_(87)_ = 2, *p* = 0.048). Finally, considering the Object AOI, the action predictor showed an effect (*χ*^2^_(1)_ = 12.04, *p* < 0.001). The Object located nearby the observer was observed longer during the Thermos action (i.e., while observing a coffee cup) compared to the Spoon action (i.e., while observing a mug; *t*_(87)_ = -3.47, *p* < 0.001).

## Conclusion

The aim of the present study was to explore how observed gaze and the implicit request for a complementary action affect motor preparation and gaze behavior in onlookers. In two experiments we specifically investigated the temporal and the spatial aspects of social engagement by manipulating the time and direction of observed gaze and request gestures.

The results from Experiment 1 showed no effect on CE when presenting a request gesture accompanied by a direct gaze to the observer, nor when the actor’s gaze was convergent with the direction of his gesture (i.e., both pointing to the target object). In the light of previous results from our laboratory on complementary actions (for reviews, see Sartori and Betti, 2015; Sartori, 2016), this finding was quite unexpected. In those studies we demonstrated corticospinal facilitation while participants observed video-clips evoking complementary gestures. However, it must be said that in all those studies only the actor’s arm was shown in the video clips, without the head. Here, we probably hindered the original effect by introducing a source of composite stimuli (i.e., the head and gaze movements) requiring complex processing. Notably, a link emerged between MEPs and readiness to interact. The more participants declared their engagement in the social interaction, the more their CE was activated. This effect was specific for the muscle potentially involved in the complementary response.

Experiment 2 was conceived to deeply investigate social motor preparation, and to disambiguate alternative explanations (see below).

### Diverting Attention DOES Affect Complementary Responses

In terms of CE, the main result of Experiment 2 was an increase of activity in the ADM muscle as soon as the actor’s arm started to move the sugar spoon toward the mug placed nearby the observer while looking straight to the her/him, compared to when he was gazing away (i.e., to the contralateral side with respect to the gesture). This finding seems to confirm the hypothesis by Reader and Holmes (2016), suggesting that the separation of gaze and body cues has adverse effects on social interaction.

In a recent TMS experiment with video clips showing similar request gestures but without head and eye cues (Betti et al., 2017), we tested the influence of distracting stimuli on complementary motor preparation. Motor activity in the muscles required to perform a complementary response (i.e., a whole-hand grasp) was impervious to the appearance of a dot diverting attention to the contralateral side of the scene with respect to the moving hand, even when it was overtly attended. This suggested that complementary motor preparation is resistant to modulation by top-down mechanisms, such as visuospatial attention. Given the social relevance of understanding the actions of others, it seems plausible that action observation areas in the brain are relatively unaffected by attention modulation. However, data from the present study in which it is the actor’s gaze that points to the contralateral side of the scene – instead of a dot – seem to suggest that social motor preparation is highly susceptible to biological cues, able to automatically capture and divert observer’s attention. This point to the existence of dedicated mechanisms for special classes of stimuli with particular biological and social significance (e.g., McKone et al., 2007).

### Early Social Engagement Enhances Corticospinal Excitability

The very fact that we found an *early* corticospinal modulation at T_1_ (i.e., at the beginning of the social request) but not at T_2_ (i.e., at the end of the request gesture) highlights two crucial aspects.

First of all, it endorses a previous finding that complementary response preparation promptly occurs and that it is time-locked to the kinematics of the actor’s arm movement (i.e., *functional shift*; Sartori et al., 2013). In that study, video-clips similar to the present stimuli were adopted and TMS was delivered at five different time points corresponding to five kinematic landmarks characterizing the observed action. The most important was an early time point, when the actor’s hand trajectory began to move toward the out-of-reach object. A TMS pulse specifically delivered at that time revealed that wrist rotation was the only salient kinematic parameter upon which observers relied to discriminate the intention of the actor, before the request was fully expressed. This confirms previous evidence unveiling the existence of heuristics based on action and gaze cues that allow intention discrimination (Sartori et al., 2011a ; Becchio et al., 2018). When an observed gesture is socially relevant, anticipatory complementary activations follow. The functional shift indicates the ability to untie the automatic tendency to mirror another’s actions and to quickly prepare a complementary response.

Secondly, the present finding suggests that the lack of effects shown in Experiment 1 was probably due to a delay in the timing of TMS stimulation. In fact, the single pulse was delivered when the actor’s arm was already stretched out toward the target object. This finding is in line with an extensive literature on action anticipation and motor resonance, showing a greater motor facilitation when observing the early stages of an action rather than the action’s conclusion (Gangitano et al., 2001, 2004; Aglioti et al., 2008). One suggested function of the motor system is to provide the visual system with predictions about the future state of an unfolding action (Blakemore and Frith, 2005; Wilson and Knoblich, 2005; Prinz, 2006) and this ability allows the selection of a suitable action from a multiplicity of possible alternatives (Bekkering et al., 2009; Sartori et al., 2013). Advance information gained while an action sequence is being observed would allow observers to interact appropriately. These findings have direct implications with regard to action representation theories as they suggest that intention attribution is sensitive to early kinematic cues (Kilner et al., 2007; Sartori et al., 2009, 2011a; Becchio et al., 2010, 2012, 2018; Manera et al., 2011).

A limitation of the current design – and of similar studies – is that a single hot spot and a single intensity were chosen for stimulating both the FDI and ADM cortical maps. Notably, the ADM’s motor representation is weaker than that of the FDI, since the little finger abduction is a relatively infrequent movement as compared to the index finger abduction that is frequently activated during pointing movements or together with the thumb to grasp and handle objects. It is then possible that setting the rMT based on ADM might have “over-stimulated” FDI (Naish and Obhi, 2015). This hypothesis would be confirmed by the fact that MEPs elicited in FDI were greater than those triggered in ADM. It is therefore possible that differences in stimulation areas, motor representations, and stimulation intensity could have influenced our pattern of results, and this is a factor to bear in mind for future studies.

### Gaze Engagement

Results from Experiment 1 suggest that gazing at the onlooker at the end of the action (i.e., Direct Gaze) increases perceived social engagement more than gazing at the object. Indeed, the more the participants felt involved in the action, the higher was the CE in the muscle required for the interaction. The joint contribution of request gestures and direct gaze maximizes the efficiency of social response preparation. As regards Experiment 2, eye-tracking data confirmed that the engaging gaze was effective in drawing observers’ attention both on the actor’s hand and on the coffee cup (Thermos condition). No effect was found when the actor’s gaze was averted, or when the request gesture was directed to the mug, an object much simpler to be handled than the coffee cup, therefore less complex in terms of affordance. This hypothesis is further confirmed by the fact that participants spent longer time observing the coffee cup than the mug. Lastly, participants observed the actor’s head for longer time during the Spoon rather than the Thermos request gesture. It is tempting to assume that the goal of the pouring gesture performed with the spoon was vaguer than the same gesture performed with the thermos. This might have induced participants to spend more time looking the actor’s head, in the attempt to clarify his intentions.

Functional imaging studies in adults have shown that activity in a cortical and subcortical network of regions defined as “the social brain network” (Johnson et al., 2005; Adolphs, 2009) – specialized in processing social information – is modulated by eye contact (Senju and Johnson, 2009). The present data confirm and extend previous literature suggesting that direct gaze not only signals that a request is relevant for the agent, but it also concurs to quickly activate the appropriate social affordance (Ferri et al., 2011; Innocenti et al., 2012; Letesson et al., 2015). Engaging in complementary interactions, in particular, is made possible by immediate understanding of another person’s intentions toward a salient object (Becchio et al., 2018) and the readiness to engage in socially meaningful situations (Di Paolo and De Jaegher, 2012). In this respect, a crucial advantage of adopting dynamic stimuli is that they approximate to a real social interaction, and this represents a step forward for the available literature on social interactions. Future studies should capitalize on the finding that social motor preparation increases with early gaze engagement (i.e., direct gaze), followed by a gaze toward the salient object for the interaction; not the other way round.

## General Conclusion

The present research suggests that social motor preparation in interactive contexts is time-locked to the combination of early kinematics cues and direct gaze. This is a novel and interesting finding and it is consistent with recent evidence showing that direct gaze contributes to the prompt activation of appropriate social affordances (Ferri et al., 2011). Complementary affordances, in particular, are a specific subcategory of social affordances referring to all those possibilities for interaction provided by others which activate appropriate motor programs aiming to bring a common goal to completion. Complementary affordances depend on a number of variables, such as: (i) the presence of salient objects necessary for an action to occur, (ii) gaze information, and (iii) the willingness to engage in a collaborative task (Sartori, 2016). The crucial role played by a salient object on the planning of an appropriate response has been widely investigated in past literature. Here, we specifically assessed the role of direct gaze in conjunction with a request gesture on a salient object. Notably, results from Experiment 2 reveal that the separation of body kinematics and averting gaze cues has adverse effects on social interaction. While further research is needed to determine the specific additional role of the third component, namely social engagement, our results are among the first to investigate the role of direct gaze on CE in social contexts eliciting complementary responses in the onlookers.

The data outlined here might contribute to shedding light on the functioning of the human motor system in social contexts and to increase our knowledge on forms of social disorders, such as autism. In computational terms, one of the long-term goals of the present and other studies (Chinellato et al., 2013) is to implement neurophysiological data into a model able to provide artificial systems, such as humanoid robots, with more advanced social skills as they interact with humans.

## Author Contributions

SB, UG, and LS designed the study and analyzed the data. GZ and SG created the stimuli and collected the data. SB, UG, UC, and LS interpreted the data and discussed the results. SB and LS wrote the manuscript. SB, UC, and LS critically revised the manuscript.

## Conflict of Interest Statement

The authors declare that the research was conducted in the absence of any commercial or financial relationships that could be construed as a potential conflict of interest.
