# Supplementary material for: Look at Me: Early Gaze Engagement Enhances Corticospinal Excitability During Action Observation
Source: Front Psychol. 2018 Aug 9;9:1408. doi: 10.3389/fpsyg.2018.01408 (PMC6095062; doi:10.3389/fpsyg.2018.01408)
Supplement: Supplementary file 1 [file Table_1.docx]

**Table 1**. Experiment 1, mean (SD) questionnaire responses.

|  |  | Condition | |
| --- | --- | --- | --- |
|  |  | **Object Gaze** | **Direct Gaze** |
| Item | **Q1** | 2.55 (1.09) | 3.52 (1.15) |
|  | **Q2** | 2.18 (1.21) | 2.45 (1.28) |
|  | **Q3** | 2.30 (1.21) | 3.55 (1.46) |
|  | **Q4** | 2.36 (1.19) | 2.79 (1.27) |

**Table 2**. Experiment 1, mean (SD) of normalized MEP amplitudes.

|  |  | Condition | |
| --- | --- | --- | --- |
|  |  | **Object Gaze** | **Direct Gaze** |
| Muscle | **FDI** | 1.13 (0.50) | 1.12 (0.53) |
|  | **ADM** | 1.10 (0.41) | 1.06 (0.38) |

**Table 3**. Experiment 2, mean (SD) of normalized MEP amplitudes.

|  |  |  | Condition | | | |
| --- | --- | --- | --- | --- | --- | --- |
|  |  |  | **Spoon Action** | | **Thermos Action** | |
|  |  | Time | **Gaze Engaged** | **Gaze Averted** | **Gaze Engaged** | **Gaze Averted** |
| Muscle | **FDI** | **T1** | 1.53 (0.84) | 1.27 (0.69) | 1.33 (0.64) | 1.27 (0.54) |
|  |  | **T2** | 1.21 (0.46) | 1.16 (0.46) | 1.26 (0.64) | 1.19 (0.54) |
|  | **ADM** | **T1** | 1.56 (0.74) | 1.29 (0.56) | 1.53 (0.72) | 1.47 (0.61) |
|  |  | **T2** | 1.36 (0.68) | 1.19 (0.68) | 1.30 (0.62) | 1.38 (0.73) |

**Table 4**. Experiment 2 (eye-tracking), mean (SD) of fixation duration within AOIs.

|  |  | Condition | | | |
| --- | --- | --- | --- | --- | --- |
|  |  | **Spoon Action** | | **Thermos Action** | |
|  | AOI | **Gaze Engaged** | **Gaze Averted** | **Gaze Engaged** | **Gaze Averted** |
| **Step A** | **Head** | 6.20 (2.46) | 6.50 (2.46) | 6.17 (2.67) | 6.06 (2.39) |
|  | **Hand** | 8.11 (2.52) | 7.66 (2.66) | 8.44 (2.37) | 8.20 (2.5) |
|  | **Object** | 0.03 (0.11) | 0.04 (0.12) | 0.05 (0.13) | 0.06 (0.12) |
|  |  |  |  |  |  |
|  |  | Condition | | | |
|  |  | **Spoon Action** | | **Thermos Action** | |
|  | AOI | **Gaze Engaged** | **Gaze Averted** | **Gaze Engaged** | **Gaze Averted** |
| **Step B** | **Head** | 4.62 (2.05) | 4.50 (2.46) | 3.92 (1.90) | 4.48 (2.22) |
|  | **Hand** | 2.87 (1.73) | 2.73 (1.98) | 2.91 (1.52) | 2.42 (1.51) |
|  | **Object** | 0.52 (0.77) | 0.52 (0.82) | 1.03 (1.50) | 0.95 (1.24) |
